# Physiological team dynamics explored: physiological synchrony in medical simulation training

**DOI:** 10.1186/s41077-025-00335-5

**Published:** 2025-03-01

**Authors:** Rafael Wespi, Andrea N. Neher, Tanja Birrenbach, Stefan K. Schauber, Marie Ottilie Frenkel, Helmut Schrom-Feiertag, Thomas C. Sauter, Juliane E. Kämmer

**Affiliations:** 1 Department of Emergency Medicine, Inselspital, Bern University Hospital, University of Bern, Bern, Switzerland; 2 Graduate School for Health Sciences, University of Bern, Bern, Switzerland; 3 Centre for Educational Measurement (CEMO) &, Department of Behavioural Medicine, University of Oslo, Oslo, Norway; 4 Faculty of Health, Safety, and Society, Psychology in Health Care, Hochschule Furtwangen University (HFU), Freiburg, Germany; 5 Department Sport Psychology, Institute for Sport and Sport Sciences, Heidelberg University, Heidelberg, Germany; 6 Austrian Institute of Technology Gmbh, Vienna, Austria

## Abstract

**Introduction:**

For researchers and medical simulation trainers, measuring team dynamics is vital for providing targeted feedback that can lead to improved patient outcomes. It is also valuable for research, such as investigating which dynamics benefit team performance. Traditional assessment methods, such as questionnaires and observations, are often subjective and static, lacking the ability to capture team dynamics. To address these shortcomings, this study explores the use of physiological synchrony (PS) measured through electrocardiogram (ECG) data to evaluate team dynamics automated and in high-resolution.

**Methods:**

A multicentre observational field study was conducted involving 214 medical first responders during mixed reality (MR) mass casualty training sessions. Participants were equipped with electrocardiogram (ECG) sensors and MR gear. The study measured dyadic PS using heart rate (HR), root mean square of successive differences (RMSSD), and standard deviation of NN intervals (SDNN). Data were collected at high frequency and analysed using dynamic time warping (dtw) to assess fluctuations in PS.

**Results:**

Findings indicate that PS varies significantly by task nature, with higher synchrony during cooperative tasks compared to baseline. Different ECG metrics offered unique insights into team dynamics. Proximity and scenario conditions influenced PS, with closer teamwork leading to higher PS. Smaller sampling intervals (e.g. 5 s) provided a detailed view of PS fluctuations over time.

**Discussion:**

The results demonstrate the potential of PS as an indicator of team performance and cohesion. High-resolution monitoring provides detailed insights into team dynamics, offering high-resolution feedback that traditional methods cannot provide. The integration of physiological measures into training programmes can enhance team performance by providing objective, high-resolution data.

**Conclusion:**

This study shows that PS, measured by ECG data, is sensitive to medical team activities, offering insights into team dynamics. Different ECG metrics highlight various aspects of team performance, and high-resolution monitoring captures detailed dynamics. Further research is needed to validate these findings across diverse scenarios. This approach could improve training methodologies, resulting in better-prepared medical teams and improved patient care outcomes.

**Supplementary Information:**

The online version contains supplementary material available at 10.1186/s41077-025-00335-5.

## Introduction

The measurement of team dynamics is of great importance, particularly in healthcare, as it enables the generation of complementary feedback. Providing structured feedback, such as during a debriefing after a team training, is imperative for continuous team improvement and performance, thereby enhancing patient safety [1–3]. For this, debriefing emphasises guided reflection, allowing participants to analyse and reinforce learning, which is critical for improving team dynamics and performance [4–6]. Besides the training context, research into team dynamics is also of great interest, such as the study of factors underlying and reinforcing team performance [7, 8].

Team dynamics, as explained by team theory, encompass complex interactions and coordination processes essential for team performance, especially in high-stakes environments [9]. Frameworks like the input-process-outcome model and team adaptation theory provide insight into how teams adapt and maintain cohesion under pressure, achieving shared goals even amid changing demands [9, 10].

Traditionally, structured debriefing sessions are used to provide feedback post-training, allowing participants to analyse and reinforce learning from specific scenarios. However, real-time feedback is increasingly sought in high-stakes environments, where capturing team dynamics as they occur can provide valuable, immediate insights. PS offers potential in this regard, serving as a data-driven, objective feedback mechanism that could complement traditional methods by highlighting instances of team cohesion and coordination in real time [6, 11].

Traditional methods of assessing team dynamics and performance typically involve questionnaires or observations [12–14]. While these methods are easy to use, they are subjective and prone to bias [15]. In addition, such assessments are often retrospective and static, overlooking the dynamic nature of team dynamics that evolve over time, making it difficult to understand how outcomes or improvements were achieved [16]. In contrast, temporal methods for measuring team dynamics provide complementary insights into how team dynamics unfold and influence outcomes at different stages [17]. One prominent approach is real-time behavioural coding, which involves structured, in-the-moment coding of team interactions, allowing for immediate analysis of group dynamics and ensuring data reliability and validity in dynamic settings [18–20]. Complementing this, physiological measurement promises to reduce subjective bias and provide unobtrusive monitoring of team dynamics [21, 22]. In particular, technology-based physiological assessments can provide coaches and researchers with automated, noninvasive, and resource-efficient data for real-time insights into team dynamics [23].

Defined as the continuous assessment of team members’ physiological states (e.g. with wearables) during collaborative tasks, team physiological dynamics (TPD) focus on the physiological interactions and is related with, for example, team coordination or team performance [21]. As a possibility to move from individual team members’ data to the team level, physiological synchrony (PS) is a method to aggregate values for TPD [21]. PS is an important part of the team — and interpersonal dynamics [24], and has been studied in fields such as developmental psychology, neuroscience, and psychotherapy [25]. It involves capturing the similarity in physiological signals collected from individual team members. The alignment of these signals during interactions to PS demonstrates the complex ways in which individuals connect, extending beyond basic communication to include physiological states [26, 27]. 

High PS among team members often indicates alignment in physiological responses, suggesting a level of cohesion that can support effective collaboration, especially in tasks requiring shared focus or coordinated effort [28, 29]. However, high PS does not always equate to optimal performance; certain tasks benefit more from individual flexibility than strict synchrony [25]. PS primarily reflects implicit coordination, where team members align their actions and attention without necessarily engaging in explicit communication, making it a valuable marker of underlying team dynamics rather than a direct measure of coordination or performance [26]. Studies have shown that better group processes, such as enhanced communication, coordination, and cohesion, are linked with PS [30–32]. These processes contribute to the team’s ability to work interdependently, adapt to changing demands, and effectively manage shared tasks, particularly in high-stakes environments [33, 34]. Studies have shown that PS in teams, such as of paramedics, correlates with better social coordination and group processes [33]. Furthermore, Mønster et al. demonstrated that PS among team members represents elements of team dynamics, such as cohesion and adaptability, particularly in cooperative tasks [29].

An important question in research on PS is as follows: What contributes to PS? Past research has revealed that, for example, common interpersonal processes, interpersonal interdependence, conditional demands, and simultaneous movements may contribute to PS (for a review, see [25]). Yet, the role of proximity between team members has not been sufficiently explored as most studies on PS have used static settings without full-body movement, which excludes the exploration of this additional variable by design [35, 36]. Assessing proximity, however, and exploring its relation to PS seem promising as it can be automatically captured during team training using appropriate technology and is thus more feasible than classical observational approaches such as video analysis or real-time observations (e.g. [37]). Therefore, the current study investigates the influence of proximity on PS.

PS is usually calculated for predefined time windows or events, resulting in a single PS value over the whole period [27, 38]. This approach is valuable for precise questions, defined time frames, and specific team tasks. However, it may be less suitable for timely high-resolution exploratory or process analyses. In order to enable a more detailed analysis of team dynamics and provide more detailed feedback on, for example relevant team behaviours, it would be beneficial to analyse the progression of synchrony over time. However, the potential of ongoing data has not yet been sufficiently explored [39]. Indeed, the majority of past work on PS in teams has compared different time windows of different events or similar with each other [24, 33, 40], providing an overview of, e.g., an entire training session but not of the course of PS dynamics within a specific situation. To assess PS, different metrics are available such as heart rate or heart rate variability [39]. Thereby, different metrics may be sensitive to different aspects of team dynamics, as they measure different physiological mechanisms, so it is important to distinguish between them:

Heart rate (HR) reflects the frequency of heartbeats and serves as a fundamental indicator of cardiac function, assessing overall workload and cardiovascular fitness. It typically increases during sympathetic activity (e.g. fight or flight) and decreases during parasympathetic activity (e.g. relaxation) [41, 42]. Heart rate variability (HRV) metrics offer deeper insights into autonomic nervous system modulation [43]. Two important metrics are as follows:

1. Root mean square of successive differences (RMSSD), which focuses on short-term variability primarily influenced by such as parasympathetic activity [44, 45].
2. Standard deviation of NN intervals (SDNN) reflect overall HRV, encompassing broader autonomic regulatory mechanisms [42].

Therefore, RMSSD was prioritised in this study as a primary metric, while SDNN was included for comparison and exploration purposes. The compilation of these metrics provides a comprehensive assessment collection. Furthermore, these three metrics demonstrated that the same psychophysiological activation was observed in simulation training and in real-life situations [46]. As a result, they serve as a foundation for the research of physiological team dynamics.

This study serves as an exploratory analysis to determine whether PS patterns can be observed and assessed within dynamic team-based medical training. Research suggests that PS may reflect underlying team processes like coordination and cohesion, which are critical in high-stakes environments [28, 34]. Unlike studies that link PS levels directly with performance outcomes, such as Mønster et al. [29], our focus here is strictly on assessing PS without making judgements about whether synchrony correlates with team performance, and without comparing different electrocardiogram (ECG metrics). In the context of ultra-short-term HRV measurements, RMSSD is widely recognised for its stability, even in intervals as short as 30 s [47]. Conversely, SDNN has shown limitations in very short recordings due to its sensitivity to lower frequency oscillations, making it less reliable under 2 min [48]. Previous studies have largely omitted to compare different ECG metrics within studies to explore the different insights they might offer (for an exception, see Elkins et al. (26)). Therefore, the aim of this study is to investigate different metrics of PS offer, namely HR, RMSSD, and SDNN, during a medical team training session. The following aims will be tested for each of the three metrics:

1. As a proof of concept, an initial investigation was conducted whether PS differed depending on the nature of the task, more specifically during a cooperative medical team task with a shared goal vs. a baseline condition where the trainees did not interact.
2. The impact of team member proximity, scenario type, and the interaction of scenario and scenario order on PS during a medical team training over time was explored. (Aim 2.1) Since little is known about ongoing measures of PS in this area, different time frames were used and compared to each other. (Aim 2.2)

The aims were addressed in a mixed reality (MR) team training, which is a digital representation of a simulation training where one moves with one’s physical body in a virtual environment in combination with tangible elements [49] and is therefore a valuable tool for medical team training with a number of benefits. The technology enables the rapid transition between various scenarios and, e.g., the tracking of trainees’ movements.

## Methods

*A multicentre observational study was conducted* to examine the physiological dynamics of medical teams during an MR mass casualty training [50] with a convenience sample, employing high-frequency longitudinal data collection.

### Participants

A total of 214 medical first responders participated in the multicentre training, which took place in Vienna, Heidelberg, Östersund, Ranst, and Madrid (149 identified as males, 64 as females; *M*_age_ = 39 years; *M*_*j*ob experience_ = 13.3 years). Individuals with pacemakers were excluded from the data analysis (*n* = 6), but they participated in the training, and respective teams were analysed without dyads including these trainees. Ethical approval was granted by the Faculty of Behavioral and Cultural Studies Ethics Committee at Heidelberg University, Germany (approval number: AZ Beu 2023 1/1). All participants provided written informed consent.

### Materials

The MR system (Refense AG, Freienbach, Switzerland) employed an HTC VIVE Focus 3 (HTC Corporation, Taoyuan City, Taiwan) headset and a video optical system for full-body tracking, accurately capturing movement with 30–40 cameras (NaturalPoint, Corvallis, USA). The physical space allowed a 10 × 10 m area where participants could move freely, enhancing the immersive nature of the training. Manikins equipped with trackers allowed for realistic interactions, including pulse checks and triage (for details, see Zechner et al. [50]). ECG monitoring was conducted using three lead ECG sensors, capturing data at a 1000-Hz sampling rate (Bittium Inc., Oulu, Finland).

### Procedure

**Fig. 1:**
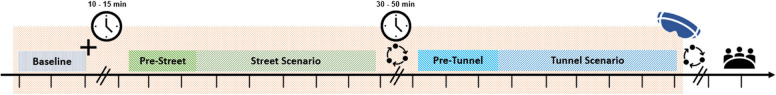
Timeline of the MR training protocol. The timeline begins with a 2-min baseline period where participants stood still, focusing on a cross to establish physiological baselines. The pre-street and pre-tunnel stages, each spanning 2 min, serve as preparatory phases without specific tasks, leading into the main street and tunnel scenarios. Each scenario was followed by a hot wash. After the second hot wash, there was a break followed by a full debriefing. Interruptions between stages are shown by diagonal lines with clocks showing approximate durations. The order of the street and tunnel scenarios was random. All activities happened in a MR environment

Upon arrival, participants were equipped with ECG electrodes followed by MR gear, and three to four participants were randomly assigned to teams for performing two first-triage training scenarios in random order. Baseline physiological data were collected, while participants stood still and fixated on a cross for 2 min (“Baseline” in Fig. 1), followed by a 2-min preparation phase allowing free movement without specific tasks (“pre-street”/ “pre-tunnel”). The scenarios simulated a bus crash mass casualty incident involving 21 injured individuals (two of them were the mannequins) and was either situated on a street or in a tunnel (“street scenario”/ “tunnel scenario”).

Instructors, who were trained medical simulation professionals with an additional 1-day VR training course, facilitated the sessions but did not intervene in the scenarios, allowing participants to organise and execute their tasks independently.

The team’s task was to be the first ambulance to arrive at the scene of an accident and perform first triage on the 21 casualties which were spread across the MR area. Participants needed to organise roles within the team, request additional support, and coordinate communication and movement to triage all injured individuals as quickly as possible.

Participants were expected to demonstrate effective teamwork by dividing roles, conducting accurate triage based on injury severity, and coordinating communication within the team to address each casualty promptly. Specific learning objectives included efficient task organisation, correct application of triage procedures, and purposeful communication to relay information to the medical commander, aligning with standard emergency response protocols.

Each scenario lasted about 8–9 min and was followed by a short debriefing (“hot wash”) and a break. Following the second hot-wash session, a comprehensive debriefing was conducted. This process encouraged participants to reflect on their actions, analyse team performance, and discuss key takeaways for improvement. Instructors provided constructive feedback on decision-making, teamwork, and adherence to protocol. Detailed guidelines for both the hot-wash and debriefing sessions are available in Appendix 1 (for details, see Zechner et al. [50]).

### Data preparation and analysis

The pre-processing steps included calculations of the three ECG metrics and determination of the distance between team members using tracking data from MR equipment. HR was calculated using a 30-s moving average. For the SDNN and RMSSD metrics, calculations were performed over a 90-s period. To assess PS, we analysed the degree of similarity in the ECG metrics of all pairs of members per team using the dynamic time warping (dtw) algorithm [51], thus resulting in three values for three-member teams and six values for four-member teams. This algorithm calculates the distance between two signals and has been found to provide a more robust and flexible analysis than Pearson correlations for calculating PS [52]. Lower dtw scores indicate that the physiological patterns of team members were more similar, suggesting higher levels of synchrony. Conversely, higher dtw scores indicate lower levels of synchrony [53]. A *p*-value of less than 0.05 was considered significant.

Proximity was calculated as the Euclidean distance between each pair of trainees in a team averaged for respective sampling intervals, where higher values indicate a larger distance between trainees. Further detailed methods can be found in Appendix 2.

#### Aim 1: Task-dependent physiological synchrony

To investigate whether PS changed depending on the nature of the task (i.e. cooperative task with a shared goal vs. baseline condition), PS per dyad within each team was determined for the baseline, the pre-scenario, and the scenario phases, respectively. Metrics of HR, RMSSD, and SDNN were used. Specifically, we calculated average dtw values per second per dyad, averaged them per phase (baseline, pre-scenario phases, and scenario phases), and compared the averaged values per phase using Wilcoxon signed-rank tests. We adjusted the results for multiple comparisons using the Benjamini–Hochberg procedure [54]. The effect sizes were reported using the rank-biserial correlation r. According to Cohen’s guidelines, values around 0.1, 0.3, and 0.5 indicate small, moderate, and large effects [55].

#### Aim 2: Influencing factors on physiological synchrony over time

We used linear mixed-effects models (LMEs) to examine how proximity, scenario type, and the order of scenarios affected PS during the cooperative team tasks (i.e. scenarios) over time. We built the models starting with including only random effects, namely group, dyad, and number of sampling intervals, followed by iteratively including fixed effects (Barr et al., 2013), namely proximity, scenario type, scenario order, and the interaction between scenario and scenario order as independent variables. The best-fitting model was chosen based on the results of an ANOVA. Dtw values based on HR, RMSSD, and SDNN and for different sampling intervals (5, 10, 15, 30, 60, 90, and 120 s) were used. The lower limit of 5 s was necessary to apply the dtw algorithm, providing enough ECG data points for meaningful comparisons within each dyad [56, 57]. Studies suggest that short windows in dtw applications enhance temporal resolution and minimise data distortion, critical for analysing physiological synchrony dynamics [58]. The upper limit of 120 s was chosen to align with the natural fluctuations of the training tasks and to ensure the relevance of the training to its dynamic nature. See Appendix 2 for more details.

## Results

### Missing data report

From the dataset comprising ECG measures of 214 participants in 60 teams, 6 ECG recordings were excluded due to technical issues. Moreover, the position data for 14 out of 120 scenarios for complete teams were missing due to technical issues. Thus, 208 participants with ECG data and 206 with position data in 60 teams were included in the data analysis.

#### Aim 1: Task-dependent physiological synchrony

##### HR

**Fig. 2:**
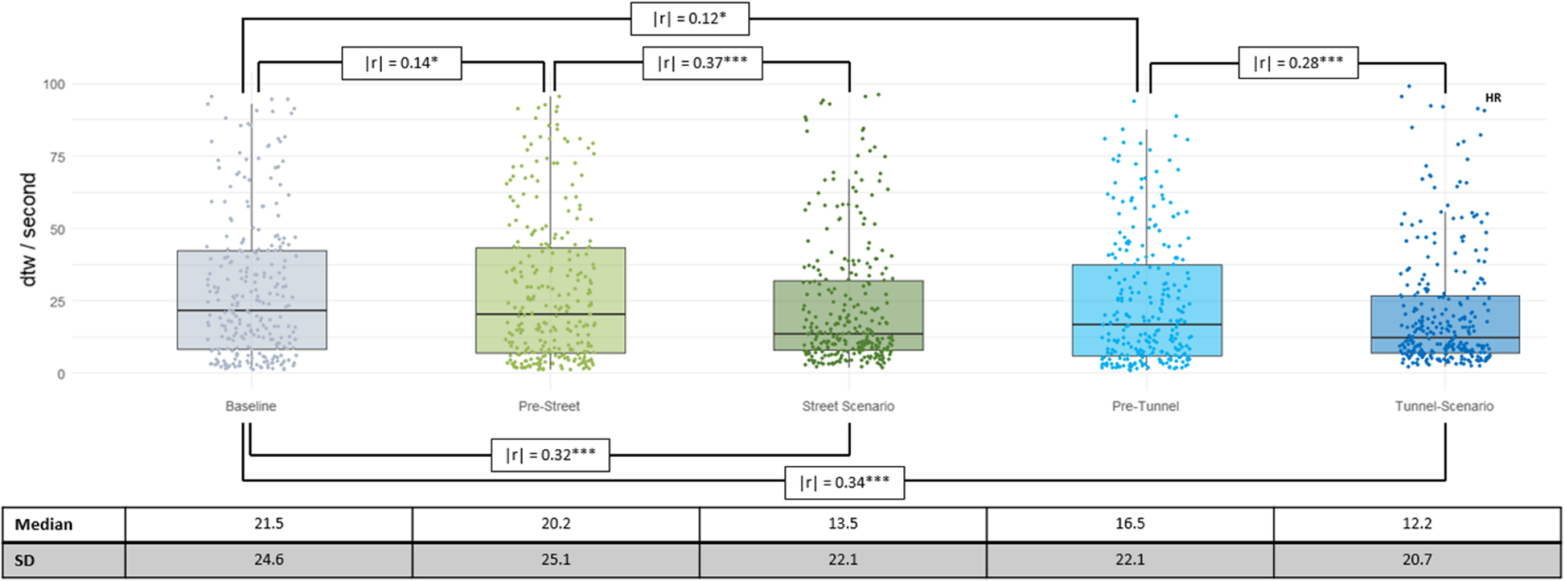
dtw/second values based on HR per phase, with raw data in the background. Additionally, significant effect sizes for comparisons between phases are shown, using | r |. The significance of the differences is Benjamini Hochberg corrected and denoted by asterisks. * indicates *p* < 0.05, and *** indicates *p* < 0.001. For better readability, the median and standard deviations (SD) of the dtw values are listed below the graph

When measuring PS based on HR per phase, we observed higher dtw values, implying less PS, in the baseline phase than in the pre-phases. Wilcoxon signed-rank tests revealed that these differences were significant and of small effect size (see Fig. 2).

Similarly, PS were lower at baseline than in the scenario phases and lower in the pre-scenario phases than in the corresponding scenario phases, all of moderate effect size.

##### RMSSD

**Fig. 3:**
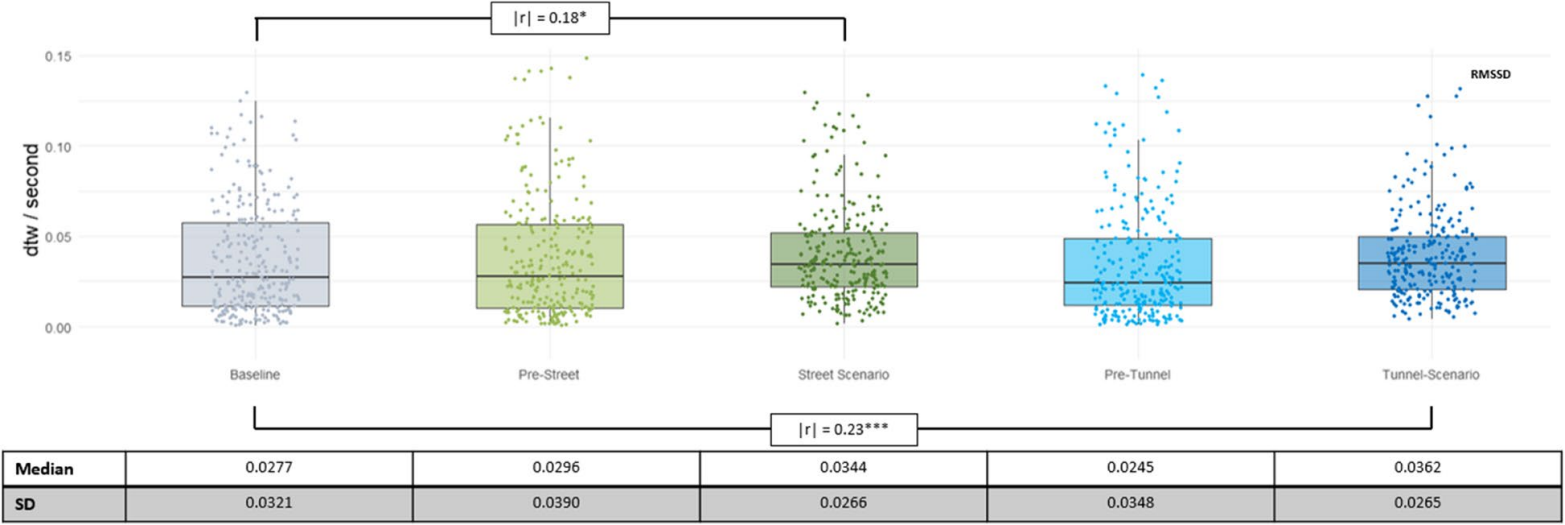
dtw/second values based on RMSSD per phase, with raw data in the background. Additionally, significant effect sizes for comparisons between phases are shown, using | r |. The significance of the differences is Benjamini Hochberg corrected and denoted by asterisks. * indicates *p* < 0.05, and *** indicates *p* < 0.001. For better readability, the median and standard deviations (SD) of the dtw values are listed below the graph

Conversely to the HR results, the analyses of PS based on RMSSD data revealed lower PS in the scenarios than in the baseline, with differences of small effect size. No other differences were observed (see Fig. 3).

##### SDNN

**Fig. 4:**
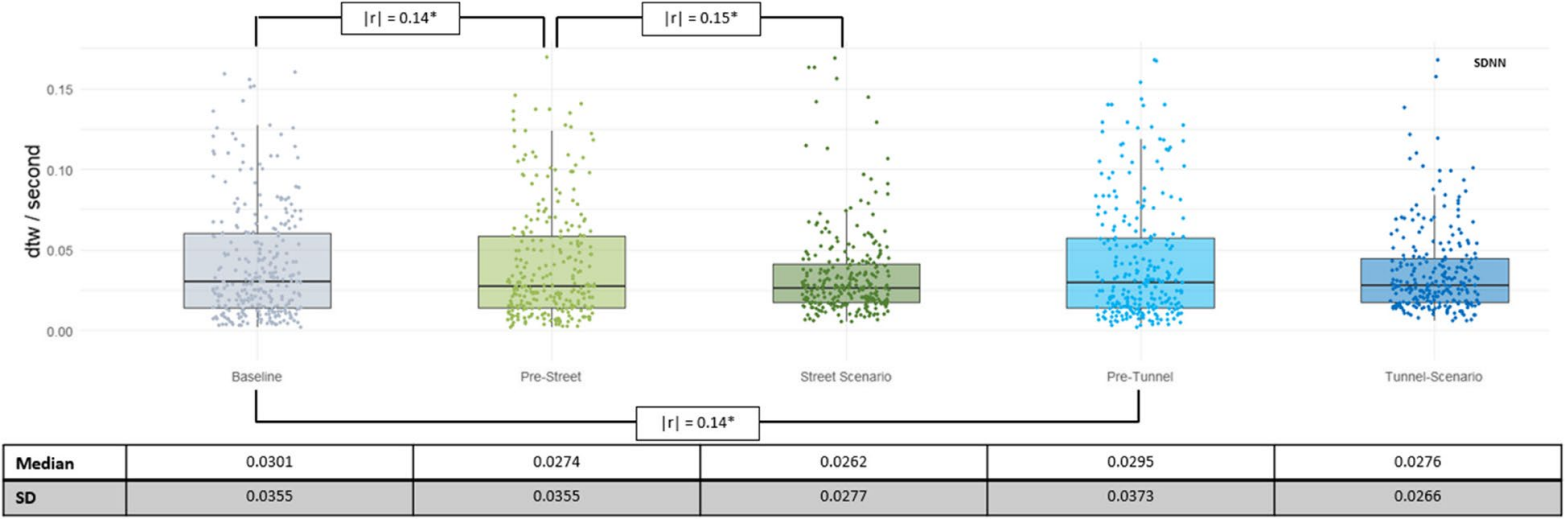
dtw/second values based on SDNN per phase, with raw data in the background. Additionally, significant effect sizes for comparisons between phases are shown, using | r |. The significance of the differences is Benjamini Hochberg corrected and denoted by asterisks. *Indicates *p* < 0.05. For better readability, the median and standard deviations (SD) of the dtw values are listed below the graph

The analyses of PS based on SDNN revealed a pattern similar to the HR results. PS was higher in the pre-scenario phases than in the baseline (as indicated by lower dtw values). Moreover, PS in the street scenario was higher than in the corresponding pre-phase, but not in the tunnel scenario (see Fig. 4). Effect sizes were small.

#### Aim 2: Factors influencing physiological synchrony over time

##### Aim 2.1: Influencing factors

The ANOVA revealed that the best-fitting LME for a sampling interval of 5 s included random intercepts for group, dyad and number of sampling intervals, and fixed effects for proximity, scenario type, scenario order, and the interaction between scenario and scenario order. Taking this LME, the analyses of PS based on HR revealed a positive effect of distance on dtw values, namely with increased distance resulting in a reduction in the PS of the respective dyad (as indicated by higher dtw values). This effect was found across all three metrics, although the values exhibited some variation from metric to metric. For further details, see Table 1 or Appendix 3.

**Table 1:**
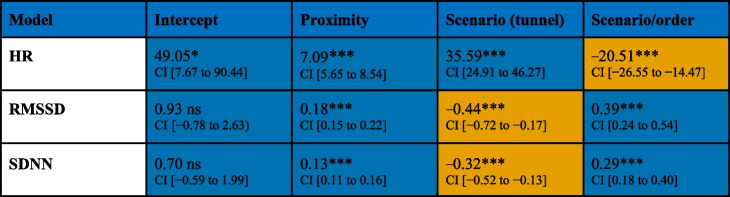
Model parameters for the influence models on PS

The results for all three ECG metrics were mapped for the sampling interval of 5 s. The parameters with a positive value are coloured blue, and those with a negative value are coloured orange. *HR* heart rate, *RMSSD* root mean square of successive differences, and *SDNN* standard deviation of NN intervals, *CI* confidence intervals.

*ns* = *p* > 0.05

*p ≤ 0.05

***p ≤ 0.001

In addition, analyses revealed an effect of scenario type, qualified by an interaction effect of scenario type × scenario order on dtw values, indicating that, in the tunnel scenario, PS based on HR was lower than in the street scenario (higher dtw values) but only if the tunnel scenario was experienced second. Opposite effects were found if PS was measured based on HRV (RMSSD and SDNN), namely that PS was higher in the tunnel scenario (lower dtw-RMSSD and SDNN values) than in the street scenario but only if the tunnel scenario was experienced second.

In this initial exploratory analysis, we focused on PS metrics to assess if they reveal meaningful patterns in team dynamics. Although additional data on control variables such as stress, age, and familiarity were collected, these were not included in this preliminary model to allow a foundational exploration of PS metrics. Future analyses will examine these control variables in relation to PS.

##### Aim 2.2: Model stability over sampling intervals

The significance of these influencing factors was then assessed across different sampling intervals (10, 15, 30, 60, 90, and 120 s) to determine their stability over time:

- At shorter intervals (with up to 10 s for RMSSD, 15 s for SDNN, and 30 s for HR), results remained stable, that is, similar effects as with 5-s intervals (see above) were revealed.
- At intermediate intervals (at 15 s for RMSSD, 30 s for SDNN, and 60 s for HR), the effect of scenario type began to fade (in HR also scenario order).
- At the longest intervals examined (starting at 60 s for each metric), the effects of scenario type and scenario order disappeared, with only proximity showing a consistent influence on PS up to 90 s for RMSSD and SDNN and 120 s for HR.

Specifically for each metric, the model stability remained as follows (see Table 2):

- HR’s responsiveness to the factors studied lasts for up to 30 s.
- RMSSD and SDNN show a more rapid decline in the stability of these effects, with a noticeable drop after 10 and 15 s, respectively.

**Table 2:**
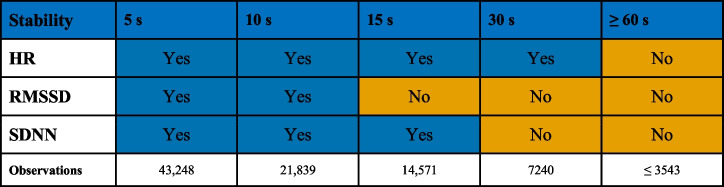
Stability of all factors in the model when they remain significant

Yes means all factors remained significant, and no means at least one factor did not remain significant. The columns for 90 and 120 s are not displayed as their stabilities are congruent with the column for 60 s. Observations represent the number of sampling intervals used for the calculation. *HR* heart rate, *RMSSD* root-mean-square of successive differences, and *SDNN* standard deviation of NN intervals

##### Illustrative example

This part provides an illustration of the potential of TPD in medical simulation training to reveal insights into previously “invisible” team dynamics. It is intended to assist readers in comprehending this approach by demonstrating how synchrony values across different metrics are depicted and seem to covary with task switches.

**Fig. 5:**
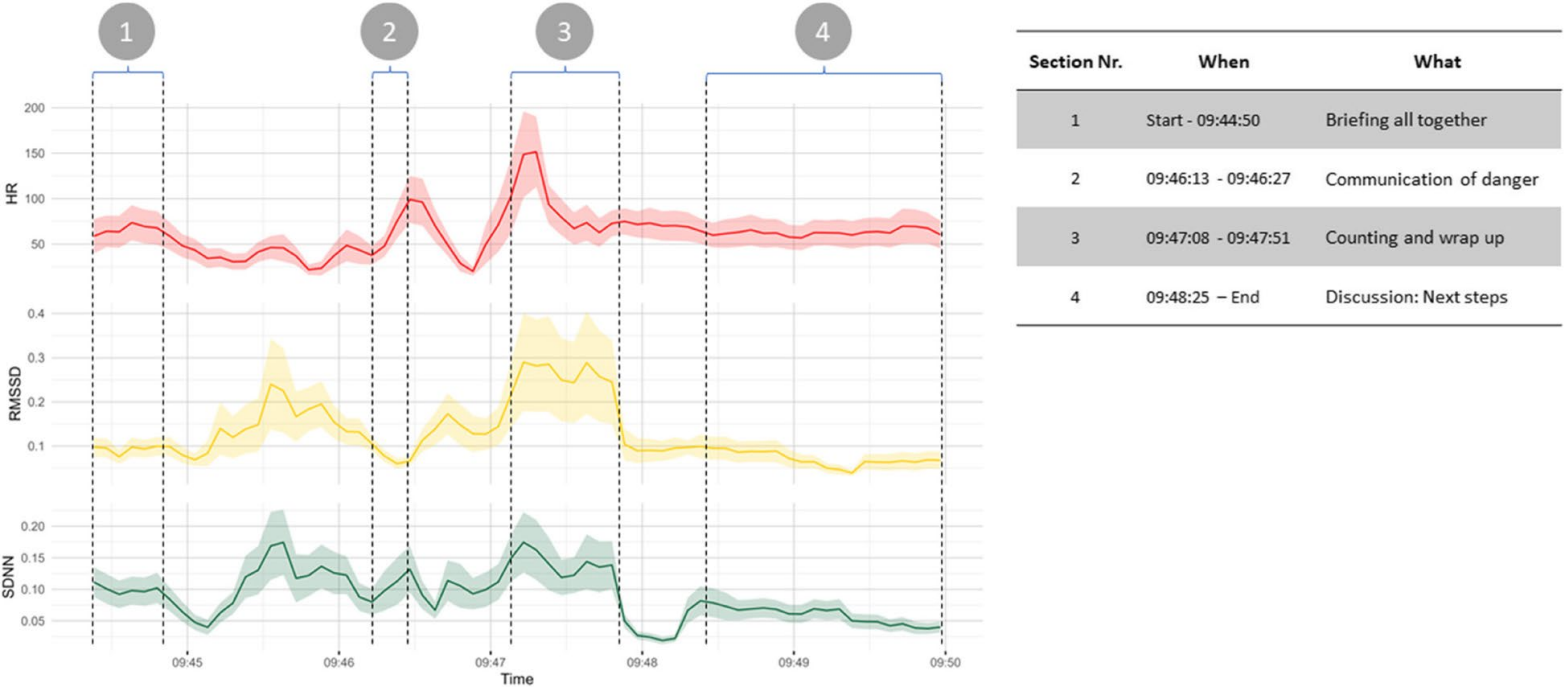
Time series of averaged PS metrics — HR, RMSSD, and SDNN — for a medical team during training. The shaded areas represent standard errors. The table on the right summarises these events. Vertical dashed lines indicate key events labelled with circled numbers, as represented in the table on the right side. *X*-axis is the time of training represented in hours and minutes. *HR* heart rate, *RMSSD* root mean square of successive differences, and *SDNN* standard deviation of NN intervals

As an example, Fig. 5 illustrates the dynamics of synchrony values across different metrics for an entire team. The plot presents group means and confidence intervals for dtw values over time, with an interval duration of 5 s. Fluctuations in synchrony metrics indicate different levels of physiological alignment between different dyads. Higher dtw peaks indicate lower PS. Three PS metrics are depicted: HR in red, RMSSD in yellow, and SDNN in green. To explore how different task demands affect team synchrony, we identified different phases of the team task by analysing the corresponding training video recording. Thereby, the timestamps of the interactions in which all trainees were involved were recorded and tasks classified. Figure 5 shows the four identified tasks in which all trainees shared their attention, namely (1) when taking part in the briefing at the beginning of the scenario, (2) when the instructor called out a danger, (3) when the team counted patients per triage category and wrapped up, and (4) when the team discussed the next steps. It can be seen that, for example, during “communication of danger” (“Methods” section), there is a marked desynchronisation in HR and SDNN, while there is a constant level of high PS during the final discussion (“Discussion” section).

## Discussion

This study aimed to explore PS within a medical team training context.

Specifically, the research had two primary aims. Firstly, it sought to assess how PS differs across different training phases and tasks. Secondly, it aimed to explore the impact of various influencing factors, including proximity, scenario type, and the scenario order, on team synchrony over time. These aims were investigated using different heart metrics, namely, HR, RMSSD, and SDNN. Although PS has the potential to serve as a valuable real-time feedback tool in team training, this study did not focus on PS in that capacity. Instead, our primary aim was to explore PS as a measure of team cohesion and implicit coordination. While feedback applications were outside the scope of this study, PS could nonetheless be developed as a structured, objective feedback tool to provide immediate insights into team dynamics during training.

Our findings indicate significant differences in PS depending on the nature of the task. Specifically, higher synchrony was observed during cooperative team tasks with HR and SDNN compared to more uncoordinated tasks. This is in line with previous research that has shown that tasks that require active coordination increase PS, as they require synchronised efforts and shared mental representations [59]. Additionally, proximity and the specific conditions of the training scenarios were found to influence synchrony during the cooperative team task, with proximity consistently enhancing PS.

These findings align with team dynamics theory, which emphasises the role of cohesion and shared mental models in promoting coordination and adaptability, especially in high-stakes environments [9]. The observed increase in PS during cooperative tasks likely reflects implicit coordination, where team members’ physiological responses align without the need for explicit communication. This type of implicit synchrony is essential in scenarios that require quick, cohesive responses [60, 61]. However, it is important to note that while high PS indicates cohesion, adaptability and individual flexibility may be equally crucial in certain tasks that benefit from less rigid alignment [10].

The ensuing discussion will be divided into two main sections following the aims: “Aim 1: Task-dependent physiological synchrony”, focusing on task-dependent PS and its sensitivity to team dynamics using HR, RMSSD, and SDNN metrics, and “Aim 2: Interpretation of factors influencing PS”, exploring the interpretation of factors influencing PS, such as proximity and scenario type. This is followed by a general discussion that bridges the empirical findings with practical applications in medical team training.

### Aim 1: Task-dependent physiological synchrony

In evaluating PS during medical team training, we employed HR, RMSSD, and SDNN. Both HR and SDNN demonstrated increased PS during active team tasks in comparison to baseline and pre-scenario phases. This suggests that they are sensitive to changes in team dynamics, which is consistent with previous work (e.g. Elkins et al. [26] or Mønster et al. [29]).

Yet, the effect size of HR metrics was moderate, whereas that of SDNN was only small. In contrast, RMSSD displayed an opposite pattern with decreased PS in cooperative scenarios. This discrepancy may be attributed to the inherent variability of RMSSD and its distinct physiological underpinnings. Research shows that increased HR is associated with reduced HRV, as higher HR leads to less variability in interbeat intervals [62]. This relationship is due to both physiological and mathematical factors, with HRV values being more closely aligned and therefore automatically increasing PS values. These effects highlight how HR influences HRV and subsequently PS measures [62].

Results show that HR and SDNN were consistent, indicating increased team cohesion, while RMSSD varied, reflecting complex interactions. Additionally, these metrics changed over time (Fig. 5), suggesting that they represent different aspects of team dynamics. Despite these findings, there is a lack of guidance in the literature on how to interpret these differences, highlighting the need for further research [26].

HR and SDNN are consistent in detecting increased PS due to coordinated actions and shared stress responses [42]. RMSSD, which reflects parasympathetic activity, varies more during high-stress tasks, resulting in reduced PS [63]. While the exact reasons for these discrepancies are unclear, the importance of these measures in assessing team dynamics remains evident [64]. Further research is essential to understand how best to use these metrics in relation to training objectives and the nature of the task.

### Aim 2.1: Interpretation of factors influencing PS

Understanding factors that influence PS can help optimise team dynamics and improve collaborative efforts. Previous studies have revealed some of these factors. For example, larger group sizes tend to show higher PS due to the increased need for coordination [28], and positive interpersonal dynamics, such as strong team cohesion and effective communication, enhance PS among team members [65]. Unlike other studies that utilised avatars independent of one’s own body or static settings, this study utilised a position-tracking MR system in which participants had to move around a room to perform a team task, allowing us to assess the effect of proximity on PS, which may can be seen as a proxy for close collaboration.

Proximity consistently demonstrated a robust positive impact on synchrony, indicating that closer physical spacing between team members enhances their PS. To the best of our knowledge, such an explicit link has not yet been established, although interpersonal relationships have already been mentioned as a relevant factor for PS in various reviews [25, 32]. It is crucial to acknowledge that while high PS can signify good team performance during collaborative tasks, lower PS may be advantageous for tasks that necessitate complementary actions or independent thinking [26].

Moreover, our analyses revealed an impact of scenario type and the interaction with order on PS, but effects varied, particularly noticeable in shorter time intervals. These effects suggest that immediate task environments and the sequence of tasks can quickly alter team dynamics, with different scenarios potentially invoking varied stress levels or learning adaptations, which, in turn, may influence PS. For example, previous research has shown that complex or high-stress scenarios may result in increased physiological arousal, which in turn affects synchrony patterns [25]. Specifically, higher levels of stress may decrease PS, as stress leads to increased individual stress responses [66].

In order to gain a deeper understanding of the processes involved in a scenario, we conducted an exemplary analysis of a sample scenario. This analysis provides perspectives into several factors that could influence PS at the scenario level (see Fig. 5):

- *Comparative analysis*: Compare dynamics of synchrony across dyads to identify which pairings have higher or lower levels of synchrony during specific situations. This can help to understand the dynamics of team interactions or identify events that have led to desynchronisation.
- *Metric-specific insights*: Different PS metrics may show different patterns of synchrony. For example, HR and SDNN may show higher synchrony during periods of high stress, whereas RMSSD may reflect more subtle autonomic adjustments.
- *Implications for training*: The findings can be used to tailor team training programmes. For example, pairs with lower synchrony might benefit from targeted interventions to improve their collaborative efficiency.

### Aim 2.2: Sampling intervals

While comparing PS measured during interactive tasks with PS during baseline conditions is a common method to isolate the impact of interaction on synchrony [25, 67], a longitudinal measurement using finer intervals/a higher temporal resolution may provide deeper insights into the time course of PS [17]. Thereby, determining the right sampling intervals is important [68]. Intervals from 5 to 120 s were used in this study.

The analysis of using different time intervals (“Aim 3: Sampling intervals”) revealed that the stability of factors influencing PS varied by metric and interval length. HR demonstrated stability for up to 30 s. In contrast, RMSSD and SDNN demonstrated stability up to 10 and 15 s, respectively (see Table 2). In comparison to “Aim 1: Task-dependent physiological synchrony”, HR, SDNN, and RMSSD show similar key values and parameters at higher temporal resolution than at lower temporal resolution, suggesting that averaging over longer periods may mask short-term synchronisation patterns, suggesting a shift towards a more averaged state of team synchrony that may not reflect rapid fluctuations. Higher resolution analysis reveals more consistent moment-to-moment physiological synchronisation among team members. The use of 5-s intervals in comparison to higher interval durations increases temporal resolution, resulting in a greater number of observations (43,248 at 5 s versus 3543 at 60 s, as shown in Table 2). This higher number of observations has the potential to affect *p*-values and, consequently, the assessment of result significance [69].

From our analyses, we would conclude that shorter intervals, particularly the 5-s minimum, seem to be optimal for detailed, dynamic analysis, as evidenced by the high number of observations and enhanced sensitivity to rapid changes in team synchrony [17]. It is also crucial to consider the temporal aspects of both the biological systems under investigation and the psychological processes to underpin their activity within that specific context [68].

### Overall discussion

PS in various measures (electrodermal activity (EDA), ECG, pupillometry, etc.) has been found to be an indicator of, for example, agreement [38], engagement [70], team performance [26–28, 71], group cohesion [72], conscious processing [73], social coordination [33], relationships [25], teamwork effectiveness [74], collective mental effort [75], and team cognitive load [76]. Yet, most of these studies have been conducted in minimal settings with minimal physical movement at a computer to avoid sensor artefacts. Given that movements can introduce artefacts or act as confounding factors, it is questionable whether the existing work can be transferred to highly physical dynamic situations such as medical team training. Here, we extend prior research by examining the impact of movement on team dynamics. Previous research indicated the potential of applying position tracking in the context of simulation training, offering an additional layer of debriefing information [77].

PS shows promise in understanding teamwork dynamics, particularly in how physiological alignment relates to effective communication, cohesion, and adaptability within teams [24, 29]. In high-stakes settings, such as emergency response teams, high PS levels could indicate well-aligned team dynamics and moments of critical coordination, suggesting synchrony as a marker of readiness and engagement [26]. Conversely, low PS might reveal communication gaps, identifying areas where teams may need additional support [21].

The relevance of PS within teamwork also opens new applications for real-time feedback systems. PS metrics could be applied in simulation-based training to provide objective insights, allowing trainers to intervene precisely when synchrony drops and potentially improving outcomes in high-pressure situations [33]. Furthermore, as PS provides a not-interfering way to observe team dynamics over time, it could help refine team-building strategies across various industries by supporting a deeper understanding of team cohesion [25].

An important consideration in using PS metrics within team-based assessments is ecological validity. While the controlled nature of simulation training allows for consistent and replicable measurements, it may not fully capture the variability present in real-life teamwork environments. Previous studies highlight that ecological validity can be affected by factors such as artificial settings, limited contextual cues, and constrained task variations, which may impact physiological responses and synchronisation [78]. Using mixed reality environments, as employed in this study, offers a promising approach by combining realistic scenarios with high measurement accuracy, thus enhancing ecological validity while retaining data consistency [49].

It is important to recognise that no single measure can comprehensively capture the overall performance of individuals or teams. To avoid the pitfalls of relying on a single inadequate criterion, it is crucial to measure both team dynamics and outcomes [79]. While automated TPD and performance measures offer valuable insights, they are not a standalone solution and should be coupled with nonautomated forms of measurement [80]. This collaborative approach should not be considered a replacement for existing evaluation methods but rather an enhancement that allows for more in-depth analysis and debriefing of team training. Physiological patterns between dyads and groups reflect meaningful changes in social processes, such as the health of interpersonal relationships [21, 25]. The utilisation of TPD as an analytical tool can provide deeper insights by measuring subconscious states alongside other factors throughout exposure [81]. By incorporating PS into performance evaluations, we could gain a more holistic understanding of team dynamics, which could significantly enhance traditional methods of team performance assessment.

### Practical considerations

One relevant aspect of using a new evaluation approach is the question of feasibility. The use of three-lead ECG measures permitted the acquisition of high-resolution PS measures of HR, RMSSD, and SDNN with minimal invasiveness. The handling of the ECG devices for recording was not time-consuming, requiring approximately 3 min per trainee. This was due to the simplicity of the placement, which could be achieved with adhesive electrodes. Furthermore, the process analysis did not necessitate calibration or similar procedures. Consequently, it was possible to monitor up to 4 team members simultaneously, with only 6 data sets being lost out of 208. The data processing was automated to a considerable extent, which would facilitate the use of the approach in real time.

At the same time, the position tracking and simulation were integrated into the MR system, which usually did not present any problems. However, it is important to note that a system of this nature comes with significant costs and may not be necessary for all applications, particularly when the calculation of, e.g., ECG-PS values is the primary aim. While this study provides preliminary evidence that PS measurements can offer insights into collaboration, further validation is needed to fully integrate these metrics into practical feedback systems. However, in the absence of a “plug-and-play” solution, further technical development, experience, and a streamlined process would be required for its use in traditional simulation training.

While ECG-based measurements of PS are minimally invasive, certain ethical and operational considerations are required for implementation [22]. In natural settings, the ECG setup remains the same, although position tracking may require alternatives such as GPS or video-based methods [37, 82]. Video recording of the training scenario can also provide valuable contextual data for later debriefing and analysis.

### Strengths and limitations

There are several limitations to this study. First, the lack of a non-MR control group means that we cannot confirm whether the PS observed in MR would also occur in real-life settings. Future research should focus on reproducing these results in real-life scenarios to validate the findings outside of VR environments. Second, while we collected additional data on control variables, including stress, age, and team familiarity, we focused this exploratory analysis on PS metrics to determine if they could reveal meaningful patterns and insights into team dynamics. Future studies in this project could further analyse these variables to assess their impact on PS. Third, the within-subjects design introduces the potential for carryover effects and increased familiarity between scenarios. Although scenario order was included as a fixed effect to control for familiarity, some residual carryover effects may still influence the results. A between-subjects design could mitigate this. Lastly, in order to maintain the process as automated and therefore as simple and straightforward as possible, we did not assess the specifics of task fulfilment, such as how, when, and what the trainees worked on together.

This approximation has limitations, as two trainees who are very close to each other can stand back to back without actually working together. This means that the effect of proximity on PS that we found may be underestimated. While proximity proved an effective measure of collaboration due to the nature of our training setup, it would be beneficial in future research to incorporate more direct measures of team interaction. Metrics such as team performance, outcomes, and communication could provide a deeper understanding of how team dynamics is related to PS. These metrics, combined with physiological data, could offer a more comprehensive model of team effectiveness in high-stakes environments.

The VR itself could be a problem, since the physical, visible, and local closeness could play a role for PS. The results of an audio listening task indicate that conscious processing of narrative stimuli and attention modulates PS based on HR, making it a potential marker for cognitive states in both healthy individuals and patients with disorders of consciousness [73]. This is particularly intriguing, given that participants completed the task independently without physical proximity to others. Other works in a VR setup demonstrated that higher PS based on EDA correlates with better task performance, thereby emphasising the value of physiological alignment in enhancing team dynamics [52]. In addition, electroencephalography (EEG)-based PS in VR can induce similar levels of inter-brain synchrony as in real-world environments [83]. In a VR context, not facing each other could reduce PS. However, verbal interaction alone can achieve synchronisation, as Gupta et al. [84] found that interaction partners aligned their blink rates while talking, even when positioned back to back. This suggests that verbal interaction may be sufficient for synchronisation, making blink rate a useful index even in virtual teams [78, 84]. This evidence suggests that the VR setup should not present a problem for deriving PS.

This study’s exploratory focus on physiological synchrony without outcome measures limits the ability to correlate PS directly with team performance metrics. Future studies should aim to integrate outcome-based metrics, such as team cohesion or task completion, to clarify PS’s role in reflecting team dynamics in high-stakes environments.

### Future direction

As the next phase in this research, correlating PS with concrete team performance measures, such as the Team Emergency Assessment Measure [13], will be essential. Exploring PS as an indicator of task efficiency, cohesion, or other outcome-based metrics can provide valuable insights into the role of physiological alignment in optimising team training. A further step should involve testing scenarios where close teamwork is crucial to assess if PS can enhance collaborative performance in more practical settings. Furthermore, correlating and linking PS with team performance outcomes, utilising tools such as the Team Emergency Assessment Measure tool (Cooper et al. [13], validated for VR by Wespi et al. [85]), will facilitate a more profound comprehension of the interrelationship between performance and team dynamics. The combination of different measures and their respective metrics will further refine these correlations [71], which could also be achieved in VR, given the tool’s capacity to facilitate eye tracking or other measures for data collection during simulations [86]. Finally, it is crucial to consider the needs and preferences of individual trainees and take necessary measures to ensure data protection.

### Practical applications and implications for medical team training

The findings of this study highlight promising applications of PS in medical team training, especially for providing real-time feedback to enhance team performance. Traditional team training in medical contexts often involves debriefing sessions post-scenario, which improves communication, coordination, and role clarity—key factors in patient outcomes and team efficacy [14, 79]. Implementing PS metrics in real-time could provide automated, objective, high-resolution feedback on team synchrony, enabling instructors to monitor cohesion and stress responses dynamically during high-stakes situations [21, 26, 27].

The use of VR and MR in medical training expands these possibilities further by simulating real-life clinical scenarios in controlled environments. For instance, MR and VR have been applied successfully in procedural and skills-based training by enabling immersive, hands-on experiences without patient risk, as shown in studies on acute care and invasive procedures [87, 88]. These technologies enhance teamwork skills, situational awareness, and adaptability, critical to clinical settings, by allowing repeated practice and real-time feedback in a realistic environment. Combining VR/MR with PS monitoring could improve training by helping trainers identify low-synchrony moments in real time, facilitating interventions that promote effective collaboration even under stress [89, 90].

By combining PS monitoring with simulation-based training, medical educators could adopt a more nuanced approach to team development, addressing both immediate and long-term training needs across diverse medical scenarios. This approach aligns with the shift towards data-driven and adaptive training methods, providing a more comprehensive understanding of team dynamics that benefits clinical outcomes.

## Conclusion

This study shows that PS measured through ECG data is sensitive to variations during a complex medical team task. Different ECG metrics can provide different insights, so it is important to consider the timing and processes underlying their activity in specific contexts.

Our findings suggest that high-resolution monitoring with smaller sampling intervals (e.g. 5 s) shows PS values and their courses and how factors such as proximity, scenario, and scenario order affect them. Although our observations on interactions cannot be generalised from a single example, they indicate potential for using PS as an indicator of team performance and cohesion.

In conclusion, our study contributes to the growing body of knowledge on the application of physiological measures in evaluating team dynamics. Our findings suggest that physiological synchrony could offer valuable insights into team cohesion and performance, potentially serving as a tool for real-time assessment in training environments. While promising, the application of PS metrics in broader contexts remains to be fully explored. Future research could further explore the applicability of PS metrics across various high-stakes, collaborative settings beyond medical training, providing insight into broader implications for teamwork dynamics in both clinical and non-clinical contexts.

## Supplementary Information

Supplementary Material 1. Appendix 1: “Hot wash” and “Debriefing” Trainer – Guidelines. Appendix 2: Detailed data preparation and analysis methods. Appendix 3: Detailed model analysis.

## Data Availability

No datasets were generated or analysed during the current study.

## Abbreviations

dtw: Dynamic time warping
ECG: Electrocardiography
EDA: Electrodermal activity
EEG: Electroencephalography
HR: Heart rate
HRV: Heart rate variability
LMEs: Linear mixed-effects models
MR: Mixed reality
PS: Physiological synchrony
RMSSD: Root mean square of successive differences
SDNN: Standard deviation of NN intervals
TPD: Team physiological dynamics
VR: Virtual reality
