# Supplementary material for: Physiological team dynamics explored: physiological synchrony in medical simulation training
Source: Adv Simul (Lond). 2025 Mar 1;10:5. doi: 10.1186/s41077-025-00335-5 (PMC11871673; doi:10.1186/s41077-025-00335-5)
Supplement: Supplementary file 1 — Supplementary Material 1. Appendix 1: “Hot wash” and “Debriefing” Trainer – Guidelines. Appendix 2: Detailed data preparation and analysis methods. Appendix 3: Detailed model analysis. [file 41077_2025_335_MOESM1_ESM.docx]

# Appendix 1 – “Hot wash” and “Debriefing” Trainer - Guidelines

Hotwash – card

Following questions serve as a guideline for the hot wash :

1. **Emotions**:
   - Do you feel ok?
   - Was there something stressful or problems that we should discuss directly now?
2. **Technique/operation:**
   - Did you get along with the technique?
   - Did you have any problems with the operation? Did something (technical) not work as expected?
3. **Reflection:**
   - Would you like to change something for the next run?
   - Were there any specific moments or aspects of the VR session that you found confusing or unclear?

**Debriefing guidelines** (short version)

1. **Setting the Scene:** Acknowledge the safe virtual reality environment for learning real-life scenarios and improving skills without risks. Identify weaknesses in the training system and scenarios.
2. **Description (By Trainees):** Trainees share their experiences, actions, and thoughts during the simulation to understand the scenario and uncover knowledge gaps or misconceptions.
3. **Analysis:** Guide trainees to critically analyze the virtual reality scenario, encouraging reflection on performance.
4. **Feedback on Performance:** Provide constructive feedback on actions and decisions, highlighting strengths and areas for improvement. Check for system-related issues.
5. **Summary:** Trainees share their lessons learned, reinforcing understanding and promoting peer learning.
6. **Key Takeaways and Action Plan:** Summarize key points for system improvement and additional training needs collaboratively.
7. **Encouragement and Support**: Recognize trainees' efforts and emphasize the journey of continuous development and learning for the MR training system.

**Assessment criteria**

| **Learning objectives**  *After the training the MFR’s are able to…* | **Assessment criteria**  *In the scenario MFR’s are demonstrating that they ..* |
| --- | --- |
| Organize and coordinate the work at the scene of the mass casualty incident | - Divide tasks - organise their work in an efficient way |
| Carry out a timely and correct triage (based on algorithm used) of MR patient (manikin) and proper use of triage card/color | - examine the injured in a proper way - Bring on the right triage colours |
| Identify the patient's vital parameters and decide on and demonstrate the correct actions and prioritizations based on triage algorithm used | - Takes correct medical actions:   - Chin lift?   - Tourniquet?   - Recovery position |
| Use purposeful communication with triage commander, team members, and injured people | - Communicate an accurate picture of the incident scene to the medical commander - Share important information within the team - interact with the injured people to obtain accurate information - Report the right numbers of injured people for each triage collor |
| Perform a continuous risk assessment | - regularly check the intervention zone for possible risks - anticipate on observations (take action – communicate - … ) |

# Appendix 2 - Detailed data preparation and analysis methods

## Data Preparation

To prepare the ECG-data for analysis, the first step was to detect the RR-peaks in the raw-data to facilitate subsequent examination of cardiac parameters. Subsequently, the dynamic time warping (dtw) algorithm was applied to calculate PS for different natures of tasks (Aim 1). The dtw was also calculated with different sampling intervals to compare different models (Aim 2). For the calculation of the distance between individuals, which was also part of the model, proximity was then calculated from the tracking data (Aim 2).

### RR-Peak detection

The ECG data was obtained in European Data Format (EDF format) and processed using Python 3.11. A bandpass filter ranging from 0.5 to 40 Hz with a 1000 Hz sampling rate was applied to refine the signal and pinpoint R-peaks via the “biosppy” library (version 2.1.2., Sutanto et al., 2020) Data from the first minute was excluded to eliminate initial recording inconsistencies. R-peak timestamps were computed and stored in CSV files for each session to streamline the analysis of large numbers of ECG files.

### Heart-Parameters calculation

Subsequent analysis of ECG data were assessed using R (version 4.3.1) and R Studio (version 2023.06.1) software. HR was calculated using a 30-second moving average, and HRV metrics such as SDNN and RMSSD over 90 seconds were calculated employing “dplyr” (version 1.1.3, Wickham et al., 2023), ”zoo”(version 1.8-12, Zeileis et al., 2023), and “lubridate” (version 1.9.3, Spinu et al., 2023) packages. The process involved reading CSV files, calculating RR intervals, and organising data by participants with identifiers and timestamps.

### Physiological synchrony calculation

For aim 1, PS was computed between the participants´ signals for each dyad of a team over the duration of the baseline, the preparation phases, and the team training phases, respectively, using dtw. Dtw measures the similarity of physiological patterns of dyads (Giorgino, 2009. 2022), whereby smaller values indicate closer similarity between sequences over time (i.e., higher PS). Conversely, larger values denote greater dissimilarity, implying more distinct patterns over time (i.e., lower PS; Lykken & Venables, 1971). For this, the "dtw" (version 1.23-1, ) R package was used. To ensure comparability between phases of different lengths, dtw values per second were calculated.

For aim 2, the continuous calculation of PS, the heart data were segmented into fixed intervals (5s, 10s, 15s, 30s, 60s, 90s and 120s) and dtw values were calculated per interval with the "dtw" package in R.

*Proximity Calculation*

To measure the proximity between dyads of trainees, the data from the back sensor of their MR equipment was analysed, using the “dplyr” (version 1.1.3, Wickham et al., 2023) and “lubridate” (version 1.9.3, Spinu et al., 2023) packages in R. The process involved computing proximity using Euclidean distance and recording them in a dataframe that includes time and pairwise distances.

## Data Analysis

The data analysis is structured into three sub-chapters, each aligning with the initial two aims of the study. Each of these sub-chapters encompasses the analysis conducted across all three-measurement types.

### Comparison of PS between team and control phases

To compare PS between team and control phases, we first determined whether there was a difference between control phases (i.e., baseline vs. both Pre-phases) and then compared control phases with team phases (e.g., Pre-Tunnel vs. Tunnel scenario / Pre-Street vs. Street scenario). This was carried out using the Wilcoxon signed-rank test. To report the effect size, the rank-biserial correlation *r* was calculated. It’s important to mention that *r* doesn’t indicate a direction of difference. According to Cohen's guidelines (1988), a value around 0.1 indicates a small or weak effect. A value around 0.3 suggests a moderate, while a value near 0.5 indicates a large or strong effect. The comparisons were repeated for all three cardiac metrics.The results were adjusted for multiple comparisons using the Benjamini-Hochberg procedure (Benjamini & Hochberg, 1995).

### Influencing factors

Linear mixed-effects models (LMEs) were used to investigate various influencing factors on PS. The 'lme4' package (version 1.1-34, Bates et al., 2023) and the 'lmerTest' package (version 3.1-3, Kuznetsova et al., 2020) in R were utilised for this purpose. LME models are particularly suitable for analysing complex datasets as they account for both fixed effects, representing general trends, and random effects, capturing individual variability. This analysis examined the relationship between proximity, scenario, scenario order as well as the interaction of scenario and scenario order among trainees and their impact on PS. The methodological approach recommended by Barr et al. (2013) was followed, refining models iteratively, starting with the specification of random effects, followed by the incorporation of fixed effects. Model selection was guided by ANOVA comparisons to determine the most suitable model structure (Barr et al., 2013). This approach is in line with the previous research, in which also dtw values (PS) were used for LME modelling (Wohltjen & Wheatley, 2021). This analysis was performed using sampling intervals of 5-second intervals.

### Sampling intervals

Subsequently, the analysis was extended to sampling intervals of 10, 15, 30, 60, 90, and 120 seconds to test the model's robustness across varying durations. These sampling intervals imply that the PS as well as the proximity were calculated over this period. Consequently, this also results in a difference in the number of values; the larger the interval, the fewer values for PS and distance in the model.

### Visualisations

All visualisations were done with the “ggplot2” (version 3.5.0, Wickham et al., 2024) and the “sjPlot” (version 2.8.15) packages.

**References**

Barr, D. J., Levy, R., Scheepers, C., & Tily, H. J. (2013). Random effects structure for confirmatory hypothesis testing: Keep it maximal. *Journal of Memory and Language*, *68*(3), 255–278. https://doi.org/10.1016/j.jml.2012.11.001

Bates, D., Maechler, M., Bolker [aut, B., cre, Walker, S., Christensen, R. H. B., Singmann, H., Dai, B., Scheipl, F., Grothendieck, G., Green, P., Fox, J., Bauer, A., simulate.formula), P. N. K. (shared copyright on, & Tanaka, E. (2023). *lme4: Linear Mixed-Effects Models using „Eigen“ and S4* (1.1-35.1) [Software]. https://cran.r-project.org/web/packages/lme4/index.html

Benjamini, Y., & Hochberg, Y. (1995). Controlling the False Discovery Rate: A Practical and Powerful Approach to Multiple Testing. *Journal of the Royal Statistical Society: Series B (Methodological)*, *57*(1), 289–300. https://doi.org/10.1111/j.2517-6161.1995.tb02031.x

Cohen, J. (1988). *Statistical Power Analysis for the Behavioral Sciences* (2. Aufl.). Routledge. https://doi.org/10.4324/9780203771587

Giorgino, T. (2009). Computing and Visualizing Dynamic Time Warping Alignments in R: The dtw Package. *Journal of Statistical Software*, *31*, 1–24. https://doi.org/10.18637/jss.v031.i07

Giorgino, T. (2022). *dtw: Dynamic Time Warping Algorithms* (1.23-1) [Software]. https://cran.r-project.org/web/packages/dtw/index.html

Kuznetsova, A., Brockhoff, P. B., Christensen, R. H. B., & Jensen, S. P. (2020). *lmerTest: Tests in Linear Mixed Effects Models* (3.1-3) [Software]. https://cran.r-project.org/web/packages/lmerTest/index.html

Lykken, D. T., & Venables, P. H. (1971). Direct Measurement of Skin Conductance: A Proposal for Standardization. *Psychophysiology*, *8*(5), 656–672. https://doi.org/10.1111/j.1469-8986.1971.tb00501.x

Spinu, V., Grolemund, G., Wickham, H., Vaughan, D., Lyttle, I., Costigan, I., Law, J., Mitarotonda, D., Larmarange, J., Boiser, J., & Lee, C. H. (2023). *lubridate: Make Dealing with Dates a Little Easier* (1.9.3) [Software]. https://cran.r-project.org/web/packages/lubridate/index.html

Sutanto, C. N., Wang, M. X., Tan, D., & Kim, J. E. (2020). Association of Sleep Quality and Macronutrient Distribution: A Systematic Review and Meta-Regression. *Nutrients*, *12*(1), Article 1. https://doi.org/10.3390/nu12010126

Wickham, H., Chang, W., Henry, L., Pedersen, T. L., Takahashi, K., Wilke, C., Woo, K., Yutani, H., Dunnington, D., Brand, T. van den, Posit, & PBC. (2024). *ggplot2: Create Elegant Data Visualisations Using the Grammar of Graphics* (3.5.1) [Software]. https://cran.r-project.org/web/packages/ggplot2/index.html

Wickham, H., François, R., Henry, L., Müller, K., Vaughan, D., Software, P., & PBC. (2023). *dplyr: A Grammar of Data Manipulation* (1.1.4) [Software]. https://cran.r-project.org/web/packages/dplyr/index.html

Wohltjen, S., & Wheatley, T. (2021). Eye contact marks the rise and fall of shared attention in conversation. *Proceedings of the National Academy of Sciences*, *118*(37), e2106645118. https://doi.org/10.1073/pnas.2106645118

Zeileis, A., Grothendieck, G., Ryan, J. A., Ulrich, J. M., & Andrews, F. (2023). *zoo: S3 Infrastructure for Regular and Irregular Time Series (Z’s Ordered Observations)* (1.8-12) [Software]. https://cran.r-project.org/web/packages/zoo/index.html

# Appendix 3 - Detailed model analysis


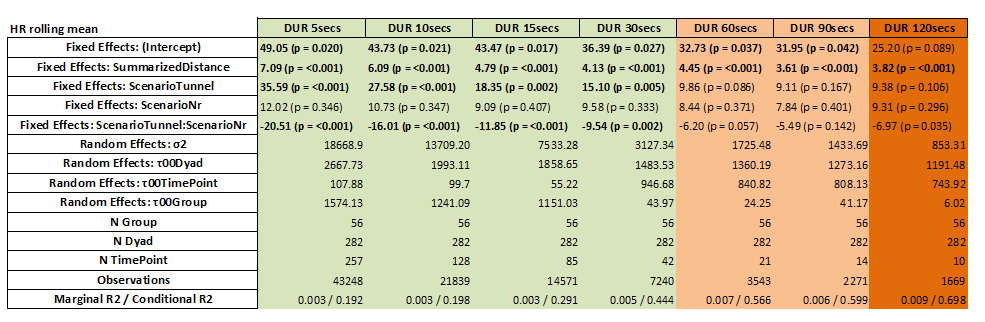


**
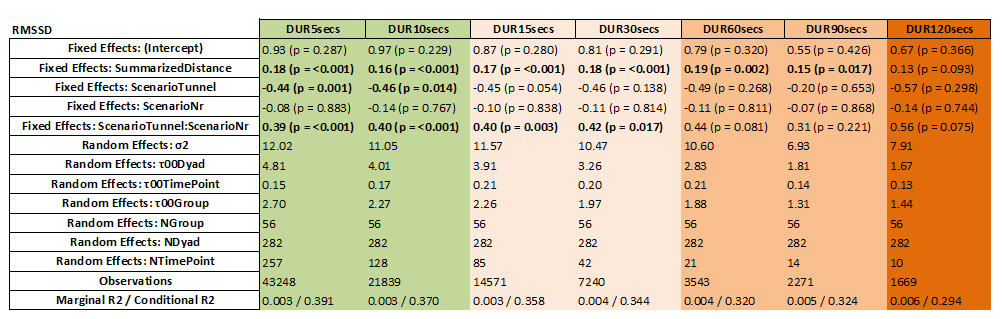
**

#
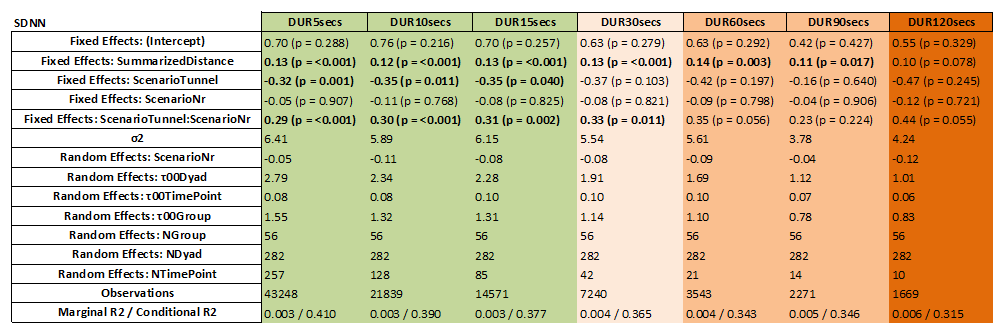


# Caption: Appendix Tables: The three tables above show the strengths of the respective influencing factors for each metric and the respective sampling interval. It can be observed that as the sampling interval increases, the *p*-values of the respective influencing factors become increasingly larger with larger sampling intervals. With longer sampling intervals, fewer influencing factors exert a significant influence on the model and thus the PS. A green background of a cell indicates that "all" influencing factors are significant; each additional colour gradation signifies that at least one other influencing factor is no longer significant. Significant values are displayed in bold font.
